# The impact of homocysteine on the risk of coronary artery diseases in individuals with diabetes: a Mendelian randomization study

**DOI:** 10.1007/s00592-020-01608-3

**Published:** 2020-10-28

**Authors:** Tian Xu, Songzan Chen, Fangkun Yang, Yao Wang, Kaijie Zhang, Guosheng Fu, Wenbin Zhang

**Affiliations:** 1 Key Laboratory of Biotherapy of Zhejiang Province, Department of Cardiology, Sir Run Run Shaw Hospital, School of Medicine, Zhejiang University, 3 East Qingchun Road, Hangzhou, 310016 Zhejiang Province People’s Republic of China; 2 Department of Cardiology, Second Affiliated Hospital, School of Medicine, Zhejiang University, Hangzhou, 310009 People’s Republic of China

**Keywords:** Plasma homocysteine levels, Coronary artery disease, Diabetes, Mendelian randomization, Causal association

## Abstract

**Aims:**

Observational studies have reported that homocysteine (Hcy) is associated with an increased risk of coronary artery disease (CAD) in individuals with diabetes, though controversy remains. The present study aimed to investigate the causal association between Hcy and CAD in individuals with diabetes.

**Methods:**

A 2-sample Mendelian randomization (MR) study was designed to infer causality. Genetic summary data on the association of single nucleotide polymorphisms (SNPs) with Hcy were extracted from the hitherto largest genome-wide association study (GWAS) of up to 44,147 individuals of European ancestry. SNP-CAD data were obtained from another recently published GWAS which included 15,666 individuals with diabetes (3,968 CAD cases, 11,696 controls). The fixed-effects inverse variance-weighted method was employed to calculate the effect estimates. Other robust methods and leave-one-out analyses were used in the follow-up sensitivity analyses. Potential pleiotropy was assessed with the MR-Egger intercept test.

**Results:**

The 2-sample MR analysis suggested no evidence of an association between genetically predicted plasma Hcy levels and CAD risk in individuals with diabetes (odds ratio = 1.14, 95% confidence interval: 0.82–1.58, *p* = 0.43) using 9 SNPs as instrumental variables. Similar results were observed in the follow-up sensitivity analyses. The MR-Egger intercept test indicated no evidence of directional pleiotropy (intercept = 0.03, 95% confidence interval: − 0.08–0.03, *p* = 0.35).

**Conclusion:**

This 2-sample MR analysis found no evidence of a causal association between plasma Hcy levels and CAD risk in individuals with diabetes.

## Introduction

Coronary artery disease (CAD) remains the leading cause of death worldwide [1–3]. Despite advances in medical and interventional treatment modalities, outcomes are still unsatisfactory in high-risk subsets of patients [4] and particularly in patients with diabetes. Coronary artery disease is a major prognostic determinant for patients with diabetes as cardiovascular death is responsible for more than 50% of mortality in patients with diabetes [5]. Therefore, much attention has been paid in the past decades to the identification of new risk factors in order to prevent CAD in individuals with diabetes. Female sex, smoking, obesity, diabetes duration, elevated systolic blood pressure and increased brain-natriuretic peptides can increase cardiovascular risk in people with diabetes [6–9].

Homocysteine (Hcy) is a sulfur-containing amino acid; its metabolism process is dependent on several nutritional and genetic factors [10]. High levels of Hcy can lead to an increased state of thrombogenicity, oxidative stress status and endothelial dysfunction [11]. Many studies have found that elevated Hcy levels are a risk factor/biomarker/predictor for developing CAD [12]. However, research on whether elevated levels of plasma Hcy correlate with an increased risk of CAD in individuals with diabetes is still relatively rare. Due to the influence of lifestyles, diet, genetics, and the environment, the incidence of hyperhomocysteinemia has gradually increased [13]. Several studies have shown that plasma Hcy levels in patients with diabetes are increased as compared with those without diabetes [14–16]. Therefore, it is particularly important to determine whether Hcy is causally correlated with an increased risk of CAD in individuals with diabetes.

The Mendelian randomization (MR) approach can effectively overcome some of the limitations of observational studies such as confounding or reverse causation [17]. Mendelian randomization has become increasingly popular for assessing and screening for potentially causal associations [18]. This study aimed to assess the causal association between plasma Hcy levels and CAD risk in individuals with diabetes.

## Methods

### Data sources

The genetic variants associated with Hcy were obtained from the hitherto largest genome-wide association study (GWAS) meta-analysis, with up to 44,147 individuals of European ancestry [19]. The corresponding genetic variants associated with CAD in individuals with diabetes were extracted from a recently published GWAS, including 15,666 individuals with diabetes (3,968 CAD cases and 11,696 controls) [20]. That study was based on UK Bio bank and all the individuals were of European ancestry. Ethics approval was not needed for this current study because it is a secondary analysis of previously published data.

### Study design

**Fig. 1:**
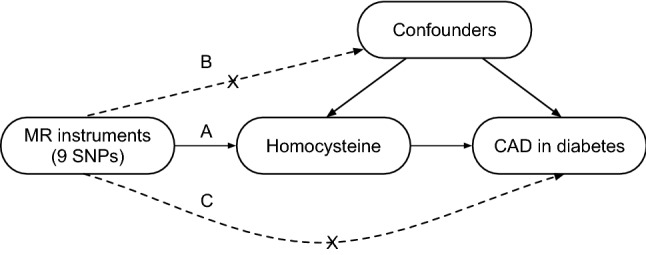
Three key assumptions of MR study. **a** SNPs must be associated with Hcy; **b** SNPs must be independent of confounders; **c** SNPs should not be directly associated with CAD in patients with diabetes. MR, Mendelian randomization; SNP, single nucleotide polymorphism; Hcy, homocysteine; CAD, coronary artery disease

A 2-sample MR study was designed to investigate the causal effect of lifetime elevated plasma Hcy levels on the risk of CAD in individuals with diabetes. The single nucleotide polymorphisms (SNPs) identified as instrumental variables for Hcy had to satisfy the following 3 key assumptions (Fig. 1): (a) SNPs must be strongly associated with plasma Hcy level, (b) SNPs must be independent of confounders, and (c) SNPs must only be associated with the risk of CAD in individuals with diabetes via plasma Hcy level [21].

### Selection and validation of SNPs

First, we selected SNPs associated with plasma Hcy levels at the genome-wide significance threshold (*p* < 5 × 10^−8^) from the Hcy GWAS. Second, we used LD-Link based on European to calculate the pairwise-linkage disequilibrium, evaluating the independence among selected SNPs [22]. When *r*^2 ^> 0.001, we removed the SNP correlated with more SNPs or with a higher *p*-value. Third, to avoid pleiotropy we looked up the remaining SNPs in the PhenoScanner and excluded those associated with other traits at genome-wide significance [23]. Finally, we calculated the F-statistic to validate the strength of each SNP, which is based on *R*^2^ (i.e., the proportion of phenotypic variance explained by each SNP).

### Primary MR analysis

In our main analysis, we employed the fixed-effects inverse variance-weighted (IVW) method to evaluate the causal effect of plasma Hcy levels on the risk of CAD in individuals with diabetes. Specifically, we generated a causal estimate for each SNP using the Wald estimator and a corresponding standard error using the Delta method. Subsequently, we obtained the overall estimate by meta-analyzing all the estimates using the fixed-effects IVW method [24].

### Sensitivity analysis and pleiotropy assessment

The random-effects IVW, penalized robust IVW, maximum likelihood, simple median, weighted median, simple mode, weighted mode, MR-Egger, and Mendelian Randomization Pleiotropy Residual Sum and Outlier (MR-PRESSO) methods were employed in follow-up sensitivity analyses. These methods may provide robust estimates against invalid SNPs [25]. In addition, a leave-one-out sensitivity analysis was performed to determine whether the overall estimate was disproportionately affected by a specific SNP. Subsequently, the MR-Egger intercept test was conducted to assess the horizontal pleiotropy and a funnel plot was generated to provide a visual inspection. All of the analyses were implemented by the “MendelianRandomization” and “TwoSampleMR” package with R version 3.6.2 (R Core Team 2019).

## Results

### SNP selection and validation

In total, we obtained 18 SNPs associated with plasma Hcy levels at a genome-wide significance level. Among them, 4 SNPs (rs12921383, rs1801133, rs2851391, rs957140) were removed because of linkage disequilibrium and 5 SNPs (rs12921383, rs1801133, rs2851391, rs957140) were excluded due to their associations with other traits at a genome-wide significance level. Finally, the remaining 9 SNPs were identified as instrumental variables in our study. The characteristics of these SNPs and their associations with Hcy and CAD in patients with diabetes are shown in Table 1. All 9 SNPs were valid (*F* > 10).

**Table 1:** The characteristics of 9 SNPs and their associations with Hcy and CAD in patients with diabetes

SNP	Nearest gene	Chr	EA	OA	EAF	*F*	SNP-Hcy association			SNP-CAD in diabetes association		
							Beta	SE	*p*-value	Beta	SE	*p*-value
rs12134663	MTHFR	1	C	A	0.2	145	0.101	0.011	2.54E–21	− 0.0176777	0.0364898	0.62806302
rs12780845	CUBN	10	A	G	0.65	56	0.0529	0.009	7.8E–10	0.00729057	0.0289842	0.80140001
rs1801222	CUBN	10	A	G	0.34	41	0.0453	0.007	8.43E–10	− 0.0258042	0.0273547	0.34551701
rs2275565	MTR	1	G	T	0.79	43	0.0542	0.009	1.96E–10	0.00873448	0.0325477	0.788423
rs234709	CBS	21	C	T	0.55	113	0.0718	0.007	3.9E–24	0.0464321	0.0267226	0.0822889
rs42648	GTPB10	7	G	A	0.6	33	0.0395	0.007	1.97E–08	0.0168946	0.0272545	0.535335
rs4660306	MMACHC	1	T	C	0.33	37	0.0435	0.007	2.33E–09	− 0.0366147	0.0283157	0.19598
rs7130284	NOX4	11	C	T	0.93	89	0.1242	0.013	1.88E–20	0.0616853	0.0509219	0.22575299
rs838133	FUT2	19	A	G	0.45	39	0.0422	0.007	7.48E–09	0.00416956	0.0276868	0.88029301

*SNP* single nucleotide polymorphism, *Hcy* homocysteine, *CAD* coronary artery disease, *Chr* chromosome, *EA* effect allele, *OA* other allele, *EAF* frequency of effect allele, *SE* standard error

### MR analyses

**Fig. 2:**
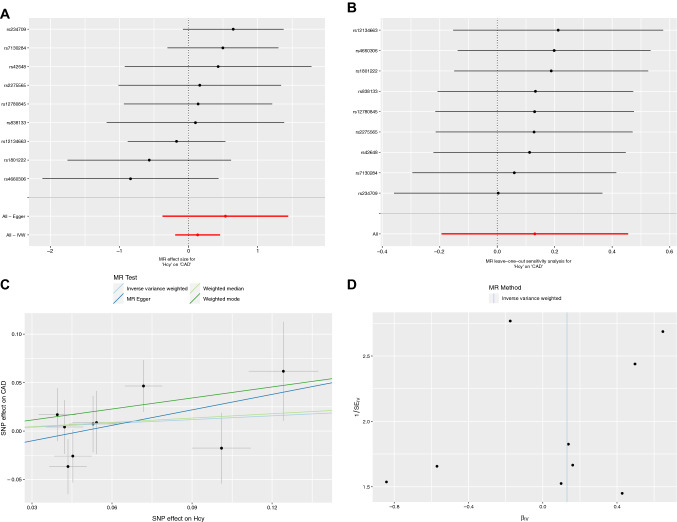
Forest plot, leave-one-out sensitivity analysis, scatter plot, and funnel plot of the association of plasma Hcy level with the risk of CAD in patients with diabetes. **a** Forest plot: the dot and bar indicate the causal estimate of plasma Hcy level on risk of CAD in patients with diabetes. **b** Leave-one-out sensitivity analysis: the dot and bar indicate the estimates and 95% confidence interval when the specific SNP is removed. **c** Scatter plot: each black dot indicates a SNP, plotted by the estimate of SNP on plasma Hcy level and the estimate of SNP on the risk of CAD in diabetes individuals with standard error bars. The slopes of the lines correspond to causal estimates using each of the different methods. **d** Funnel plot: Each black dot indicates a SNP, and the line indicates the overall estimate using IVW method. Hcy, homocysteine; CAD, coronary artery disease; SNP, single-nucleotide polymorphism; IVW, inverse variance-weighted

The fixed-effects IVW analysis suggested no evidence of an association between genetically predicted plasma Hcy levels and CAD risk in individuals with diabetes (odds ratio = 1.14, 95% confidence interval: 0.82–1.58, *p* = 0.43), as shown in Table 2 and Fig. 2a. Similar results were observed in the sensitivity analyses using the other robust methods (Table 2). The leave-one-out analysis and scatter plot also confirmed no evidence of association (Figs. 2b, c). The MR-Egger intercept test indicated no evidence of horizontal pleiotropy (Table 3), which was confirmed by visual inspection of the funnel plot (Fig. 2d).

**Table 2:** Association of plasma Hcy levels and risk of CAD in patients with diabetes using different methods

Method	OR (95% CI)	*p*
IVW (Fixed-effects)	1.14 (0.82–1.58)	0.43
IVW (Random-effects)	1.14 (0.82–1.58)	0.43
Penalized robust IVW	1.14 (0.81–1.61)	0.46
Maximum likelihood	1.14 (0.82–1.58)	0.43
Simple median	1.15 (0.70–1.87)	0.58
Weighted median	1.16 (0.75–1.79)	0.50
Simple mode	1.28 (0.62–2.63)	0.52
Weighted mode	1.46 (0.71–3.00)	0.33
MR-Egger	1.70 (0.69–4.24)	0.25
MR-PRESSO	1.14 (0.84–1.55)	0.43

*Hcy* homocysteine, *CAD* coronary artery disease, *OR* odds ratio, *CI* confidence interval, *IVW* inverse variance-weighted, *MR-PRESSO* Mendelian Randomization Pleiotropy Residual Sum and Outlier

**Table 3:** MR-Egger regression intercept

Exposure	Outcome	Intercept (95% CI)	*p*-value
Hcy	CAD in diabetes	− 0.03 (− 0.08, 0.03)	0.35

*MR* Mendelian randomization, *CI* confidence interval, *Hcy* homocysteine, *CAD* coronary artery disease

The insignificant result (*p* > 0.05) indicates no pleiotropy exists

## Discussion

This is the first 2-sample MR study to investigate the association between plasma Hcy levels and risk of CAD in patients with diabetes. This study found no evidence for the presence of a causal effect of genetically determined plasma Hcy levels on CAD risk in patients with diabetes. Elevated plasma Hcy levels may just be a biomarker for cardiovascular incidents in patients with diabetes. The findings were robust in sensitivity analyses with different statistical models.

Homocysteine, a non-protein amino acid produced by the breakdown of methionine in vivo, has been well known as far back to the 1960s [26]. Disruption of Hcy metabolism leads to hyperhomocysteinemia, which affects multiple body systems and different pathological conditions, including vascular and neurodegenerative diseases [27–29]. A direct association between plasma Hcy levels and CAD has been found in observational epidemiological studies [12]. Homocysteine could produce complex changes within the blood vessel wall [30] and may cause CAD through the following 5 pathways [31–33]: (a) vascular endothelial cell damage and dysfunction; (b) dyslipidemia; (c) promotion of the expression of inflammatory factors (e.g., the expression of tumor necrosis factor-*α* and inducible nitric oxide (NO) synthase (iNOS)); (d) impaired endothelium-mediated platelet inhibition, which enhances coagulation function and induces thrombosis; and (e) stimulation of vascular smooth muscle cell proliferation. Even mild or moderate elevation of plasma concentrations of Hcy (plasma Hcy > 16 µM) can affect coronary and peripheral arteries, eliciting vasomotor dysfunction and increased thrombosis, and consequently increased morbidity and mortality [10, 32].

Some studies have specifically investigated the association between Hcy and atherosclerosis among patients with diabetes. A prospective cohort study demonstrated that elevated levels of Hcy are associated with increased cardiovascular risk, especially in patients with diabetes [34]. A larger case–control study found that patients with diabetes and CAD had significantly higher plasma levels of Hcy than patients with diabetes and without cardiovascular damage [35]. However, controversial or negative results have been observed in other studies [36, 37]. In a clinical trial, researchers dosed Hcy in 155 subjects with type 2 diabetes and assessed whether high levels were related to chronic complications. The researchers reported that elevation of plasma Hcy levels in patients with type 2 diabetes was associated with a higher prevalence of peripheral arteriopathy; however, the study found no evidence to support the presence of a relation between Hcy and CAD [38]. Previous findings of an association between high levels of Hcy and increased incidence of CAD in patients with diabetes have been controversial. One possible explanation is that the studies’ observational designs mean sample sizes that might not be large enough to detect true associations. Moreover, in observational studies, when confounders are unobserved because they are usually unknown or unmeasured, or when the number of confounders is too large, regression methods may fail to provide unbiased estimates of the true association.

Mendelian randomization is a strategy used to determine whether a biomarker is causally involved in disease development [21]. Other studies have described MR in detail [39, 40]. Briefly, if genetic variants that robustly predict an exposure of interest can be identified, they can be used as unconfounded proxies for that exposure. Associations between the variants and the outcome of interest can thus provide evidence of causation while eliminating the problems of confounding or reverse causation. Genetic association studies are deemed as more similar to randomized clinical trials than other types of observational epidemiological studies due to MR (Mendel’s second law) [41]. Multiple GWAS have been published over the past decades, making MR a time- and cost-efficient approach [18].

In this study, we analyzed the association between plasma Hcy levels and CAD in patients with diabetes with the aid of the hitherto largest GWAS meta-analysis. We found that an increase in plasma Hcy levels did not directly lead to the occurrence of CAD in patients with diabetes. A recent study by Liu et al. [42] used the MR approach and, consistent with our findings, reported no causal relation between plasma Hcy levels and coronary heart disease or acute myocardial infarction among the general population. A previous study suggested that high levels of Hcy were related to a higher risk of stroke among general population according to the evidence on MR [43]. However, another recent study used the MR approach to confirm that the causal relation between plasma Hcy levels and stroke is limited to the small vessel stroke subtype within the general population. They found no causal relations between plasma Hcy levels and other stroke subtypes within the general population [44]. The possibility of Hcy being an independent risk factor for CAD in individuals with diabetes was unlikely. But Hcy could potentially participate in the pathogenesis of CAD in patients with diabetes. Elevated plasma Hcy levels may likely be considered biomarkers or concomitants of incident CAD in patients with diabetes. In addition, high Hcy levels observed in traditional observational studies might also be a consequence of CAD that occurred in patients with diabetes.

A strength of this study is its design (i.e., MR analysis of Hcy-related SNPs and SNPs-CAD in individuals with diabetes from large-scale GWAS). Using the 2-sample MR approach, we were able to investigate the effect of Hcy in a large sample size (44,147 individuals associated with Hcy, 3968 CAD cases, and 11,696 controls). The following potential limitations warrant discussion. First, it is difficult to completely exclude the influence of potential directional pleiotropy; namely, a genetic variant might affect the outcome via other pathways, which may lead to biased estimates. However, we ruled out as many SNPs associated with recognized confounders as possible. In addition, no evidence of a pleiotropic effect was observed in the MR-Egger intercept test, and similar results were observed in sensitivity analyses using several other models. Second, we were unable to perform a reverse analysis, because the GWAS for Hcy was not publicly available. Third, we only investigated the relation between Hcy and CAD that occurred in patients with diabetes from a genetic point of view, without considering potential environmental factors. Finally, the examined GWAS were primarily conducted in individuals of European ancestry, so the results cannot be generalized to all populations.

## Conclusion

This 2-sample MR analysis found no evidence to support the presence of a causal association between plasma Hcy levels and CAD risk in individuals with diabetes. It is plausible that simply prescribing Hcy-lowering vitamin supplementation may not decrease the incidence of CAD in patients with diabetes in clinical practice.

## Availability of data and material

The datasets analyzed in this study are publicly available summary statistics.
